# Supplementary figures and images for: Lowland extirpation of anuran populations on a tropical mountain
Source: PeerJ. 2017 Nov 15;5:e4059. doi: 10.7717/peerj.4059 (PMC5694215; doi:10.7717/peerj.4059)

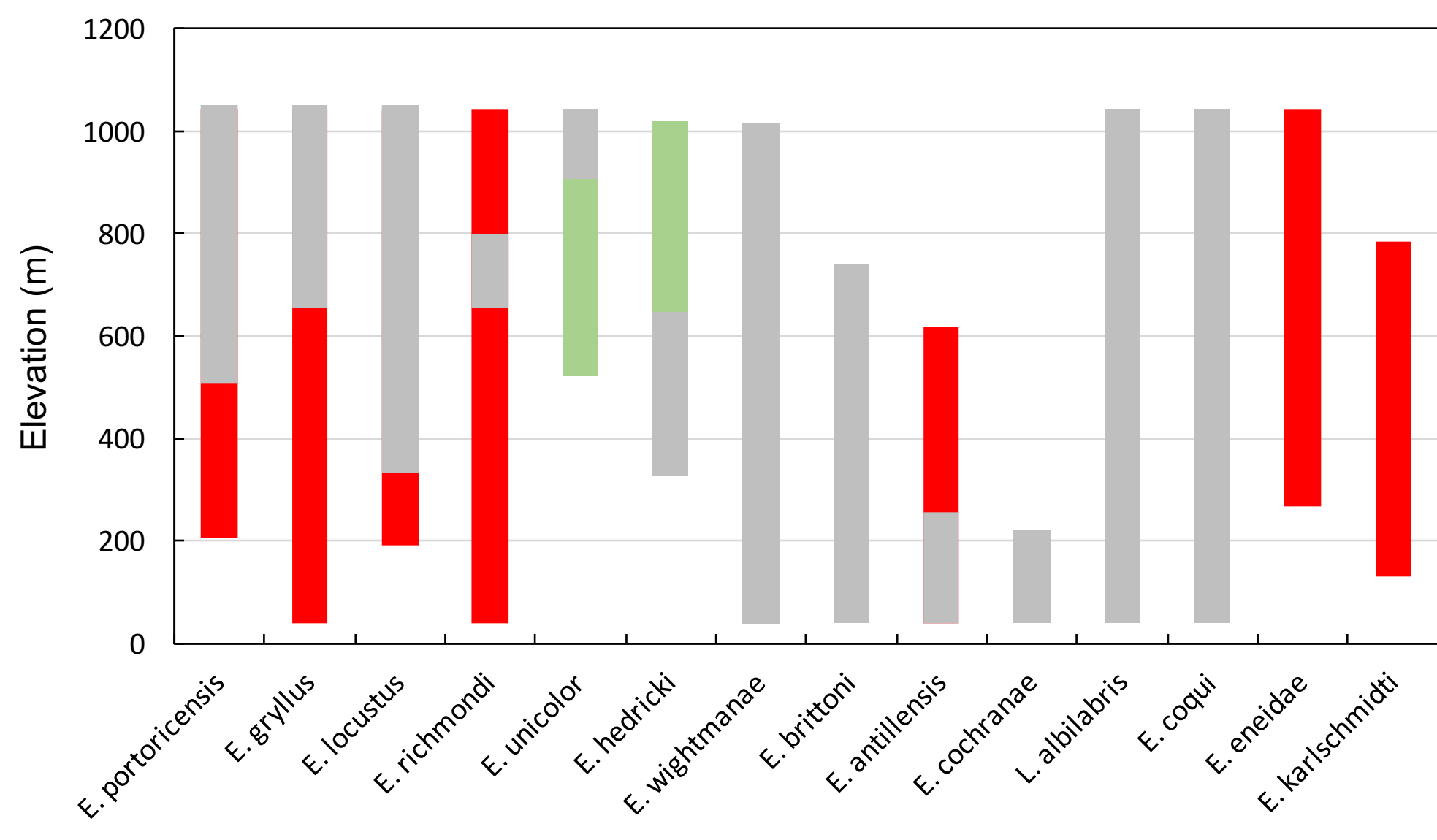

Supplement: Figure S1 — Significant shifts (>100 m) are in red for extirpations and green for colonization, while no-significant shifts are in grey. [file peerj-05-4059-s001.pdf]
