# Supplementary material for: Lowland extirpation of anuran populations on a tropical mountain
Source: PeerJ. 2017 Nov 15;5:e4059. doi: 10.7717/peerj.4059 (PMC5694215; doi:10.7717/peerj.4059)
Supplement: Table S1 — Quantitative data refers to information about the presence of the species associated with a specific georeferenced location while qualitative data refers to general information about species distribution range. [file peerj-05-4059-s002.docx]

Table S1: Summary of the historical data compilation. Quantitative data refers to information about the presence of the species associated with a specific georeferenced location while qualitative data refers to general information about species distribution range.

| **Reference** | **Quantitative** | **Qualitative** | **Detection** | | |
| --- | --- | --- | --- | --- | --- |
|  |  |  | **Visual** | **Acoustic** | **Capture** |
| (Narins & Capranica 1978) | X |  |  | X |  |
| (Van Berkum *et al.* 1982) | X |  | X |  | X |
| (Drewry & Rand 1983) | X |  |  | X |  |
| (Stewart & Pough 1983) |  | X |  |  |  |
| (Narins 1983) | X |  | X | X |  |
| (Woolbright 1985) | X |  | X | X | X |
| (Narins & Smith 1986) | X |  | X | X | X |
| (Woolbright & Stewart 1987) | X |  | X |  | X |
| (Townsend 1989) | X |  |  |  |  |
| (Schwartz & Henderson 1991) |  | X | X | X |  |
| (Woolbright 1991) | X | X | X | X | X |
| (Stewart & Rand 1991) | X |  | X | X | X |
| (Moreno 1991) | X | X | X | X | X |
| (Gonser & Woolbright 1995) | X |  | X | X | X |
| (Stewart 1995) | X |  | X | X |  |
| (Woolbright 1996) | X |  | X | X | X |
| (Joglar & Burrowes 1996) | X |  | X | X |  |
| (Woolbright 1997) | X | X | X | X | X |
| (Joglar 1998) | X | X | X | X | X |
| (Rivero 1998) |  | X | X | X |  |
| (Burrowes, Joglar & Green 2004) | X |  | X | X | X |
| (Joglar *et al.* 2007) | X | X | X | X | X |
| (Burrowes, Longo & Joglar 2008) | X |  |  |  |  |
| (Burrowes 2009) |  | X |  |  |  |
| GBIF1931-1989 | X |  |  |  | X |

**Reference**

Van Berkum, F., Pough, H., Stewart, M.M.. & Brussard, P.F. (1982) Altitudinal and Interspecific Differences in the Rehydration Abilities of Puerto Rican Frogs. *Physiological Zoology*, **55**, 130–136.

Burrowes, P. (2009) Climate Change and Amphibian Declines. *Amphibian Biology Vol 8: Amphibian Decline: Diseases, Parasites, Maladies and Pollution* (eds H. Heatwole), & J.W. Wilkinson), pp. 3268–3287. Surrey Beatty and Sons Publishers, Australia.

Burrowes, P.A., Joglar, R.. & Green, D.. (2004) Potential causes for amphibian declines in Puerto Rico. *Herpetologica*, **60**, 141–154.

Burrowes, P.A., Longo, A. V. & Joglar, R.L. (2008) Geographic distribution of Batrachochytrium dendrobatidis in Puerto Rico. *Herpetological Review*, **39**, 321–324.

Drewry, G.E. & Rand, A.S. (1983) Characteristics of an acoustic community : Puerto Rican frogs of the genus Eleutherodactylus. *Copeia*, 941–953.

Gonser, R.A. & Woolbright, L.L. (1995) Homing Behavior of the Puerto Rican Frog, Eleutherodactylus coqui. *Journal of Herpetology*, **29**, 481–484.

Joglar, R. (1998) *Los Coquíes de Puerto Rico: Su Historia Natural Y Conservación*, Primera ed. Universidad de Puerto Rico, San Juan.

Joglar, R.L., Álvarez, A.O., Aide, M.T., Barber, D., Burrowes, P.A., García, M.A., León-Cardona, A., Longo, A. V, Pérez-Buitrago, N., Puente, A., Rios-López, N. & Tolson, P.J. (2007) Conserving the Puerto Rican herpetofauna. *Applied Herpetology*, **4**, 327–345.

Joglar, R.L. & Burrowes, P. (1996) Declining amphibian populations in Puerto Rico. *Contributions to West Indian Herpetology: A tribute to Albert Schwartz* (eds R. Powell), & R.. Henderson), pp. 371–380. Ithaca.

Moreno, J.A. (1991) Status y distribución de los reptiles y anfibios de la region de Puerto Rico. *Departamento de Recursos Naturales de Puerto Ric*, 1–35.

Narins, P.M. (1983) Divergence of Acoustic Communication Systems of Two Sibling Species of Eleutherodactylid Frogs. *Copeia*, **1983**, 1089–1090.

Narins, P. & Capranica, R. (1978) Communicative significance of the two-note call of the treefrogEleutherodactylus coqui. *Journal of comparative physiology*, **127**, 1–9.

Narins, P.M. & Smith, S.L. (1986) Clinal variation in anuran advertisement calls: basis for acoustic isolation? *Behavioral Ecology and Sociobiology*, **19**, 135–141.

Rivero, J.A. (1998) *Los Anfibios Y Reptiles de Puerto Rico*. University of Puerto Rico Press, San Juan, Puerto Rico.

Schwartz, A. & Henderson, R.W. (1991) *Amphibians and Reptiles of the West Indies: Descriptions, Distributions and Natural History*. University Press of Florida, Florida.

Stewart, M. (1995) Climate driven population fluctuations in rain forest frogs. *Journal of Herpetology*, **29**, 437–446.

Stewart, M.M. & Pough, F.H. (1983) Population density of tropical forest frogs: Relationto retreat sites. *Science*, **5**, 570–572.

Stewart, M.M. & Rand, A.S. (1991) Vocalizations and the Defense of Retreat Sites by Male and Female Frogs, Eleutherodactylus-Coqui. *Copeia*, **1991**, 1013–1024.

Townsend, D.S. (1989) The Consequences of Microhabitat Choice for Male Reproductive Success in a Tropical Frog (Eleuthrodactylus coqui). *Herpetological Monographs*, **45**, 451–458.

Woolbright, L.L. (1985) Patterns of nocturnal movement and calling by the tropical frog Eleutherodactylus coqui. *Herpetologica*, **41**, 1–9.

Woolbright, L.L. (1991) The Impact of Hurricane Hugo on Forest Frogs in Puerto Rico. *Biotropica*, **23**, 462–467.

Woolbright, L.L. (1997) Local extinctions of anuran amphibians in the Luquillo Experimental Forest of Northeastern Puerto Rico. *Journal of Herpetology*, **31**, 572–576.

Woolbright, L.L. & Stewart, M.M. (1987) Foraging Success of the Tropical Frog , Eleutherodactylus coqui : The Cost of Calling. *Copeia*, **1987**, 69–75.
