# Supplementary material for: Lowland extirpation of anuran populations on a tropical mountain
Source: PeerJ. 2017 Nov 15;5:e4059. doi: 10.7717/peerj.4059 (PMC5694215; doi:10.7717/peerj.4059)
Supplement: Table S2 [file peerj-05-4059-s003.pdf]

Species	Institution	# records
E. karlschmidti	University of Kansas Biodiversity Institute Herpetology Collection	65
E. karlschmidti	Natural History Museum of Los Angeles County	2
E. karlschmidti	California Academy of Sciences	1
E.brittoni	University of Kansas Biodiversity Institute Herpetology Collection	1
E.brittoni	National Museum of Natural History	15
E.brittoni	Cornell University Museum of Vertebrates	4
E.brittoni	Texas Natural History Collection	3
E.coqui	California Academy of Sciences	3
E.coqui	Cornell University Museum of Vertebrates	435
E.coqui	University of Kansas Biodiversity Institute Herpetology Collection	170
E.coqui	University of Colorado Museum	2
E.coqui	National Museum of Natural History	37
E. antillensis	Cornell University Museum of Vertebrates	36
E. antillensis	The Museum of Vertebrate Zoology	1
E. antillensis	National Museum of Natural History	10
L. albilabris	California Academy of Sciences	1
L. albilabris	Cornell University Museum of Vertebrates	9
L. albilabris	University of Kansas Biodiversity Institute Herpetology Collection	14
L. albilabris	The Museum of Vertebrate Zoology	1
E.cochranae	California Academy of Sciences	3
E.cochranae	Cornell University Museum of Vertebrates	4
E.cochranae	National Museum of Natural History	10
E. eneidae	California Academy of Sciences	4
E. eneidae	Cornell University Museum of Vertebrates	8
E. eneidae	University of Kansas Biodiversity Institute Herpetology Collection	45
E. eneidae	Natural History Museum of Los Angeles County	4
E. eneidae	National Museum of Natural History	4
E. hedricki	Museum of Comparative Zoology, Harvard University	1
E. hedricki	Texas Natural History Collection	2
E. hedricki	National Museum of Natural History	4
E. portoricensis	California Academy of Sciences	18
E. portoricensis	C.M. Russell Museum	1
E. portoricensis	The Canadian Museum of Nature	1
E. portoricensis	Cornell University Museum of Vertebrates	75
E. portoricensis	University of Kansas Biodiversity Institute Herpetology Collection	131
E. portoricensis	Natural History Museum of Los Angeles County	39
E. portoricensis	The Museum of Vertebrate Zoology	3
E. portoricensis	University of Colorado Museum	1
E. portoricensis	National Museum of Natural History	15
E. richmondi	Monte L. Bean Life Science Museum	5
E. richmondi	California Academy of Sciences	12
E. richmondi	C.M. Russell Museum	2
E. richmondi	Cornell University Museum of Vertebrates	8
E. richmondi	University of Kansas Biodiversity Institute Herpetology Collection	65
E. richmondi	Natural History Museum of Los Angeles County	8
E. richmondi	Museum of Comparative Zoology, Harvard University	10

E. richmondi	The Museum of Vertebrate Zoology	19
E. richmondi	The Natural History Museum in London	2
E. richmondi	Texas Natural History Collection	5
E. richmondi	National Museum of Natural History	30
E. unicolor	University of Kansas Biodiversity Institute Herpetology Collection	1
E. unicolor	Louisiana State University, Museum of Natural Science	2
E. unicolor	Museum of Comparative Zoology, Harvard University	1
E. unicolor	The French National Museum of Natural History	1
E. unicolor	Texas Natural History Collection	1
E. unicolor	National Museum of Natural History	12
E. wightmanae	California Academy of Sciences	3
E. wightmanae	C.M. Russell Museum	3
E. wightmanae	Cornell University Museum of Vertebrates	16
E. wightmanae	University of Kansas Biodiversity Institute Herpetology Collection	27
E. wightmanae	Natural History Museum of Los Angeles County	3
E. wightmanae	National Museum of Natural History	11
E. locustus	Cornell University Museum of Vertebrates	14
E. locustus	University of Kansas Biodiversity Institute Herpetology Collection	166
E. locustus	Museum of Comparative Zoology, Harvard University	1
E. locustus	Texas Natural History Collection	5
E. locustus	National Museum of Natural History	18
E. gryllus	California Academy of Sciences	9
E. gryllus	C.M. Russell Museum	1
E. gryllus	Cornell University Museum of Vertebrates	9
E. gryllus	University of Kansas Biodiversity Institute Herpetology Collection	5
E. gryllus	Museum of Comparative Zoology, Harvard University	38
E. gryllus	The Museum of Vertebrate Zoology	1
E. gryllus	The Natural History Museum in London	2
E. gryllus	Texas Natural History Collection	6
E. gryllus	National Museum of Natural History	1

Address

C.M. Russell Museum

C.M. Russell Museum Complex, Great Falls, Montana, in the United States.
https://cmrussell.org/

California Academy of Sciences

CAS: California Academy of Sciences, Herpetology, Golden Gate Park, San Francisco, California 94118, USA.
http://www.calacademy.org/

Cornell University Museum of Vertebrates

CUMV: Cornell University Museum of Vertebrates, 159 Sapsucker Woods Rd, Ithaca, NY 14850, USA
http://www.cumv.cornell.edu/

Louisiana State University, Museum of Natural Science

LSUMZ: Louisiana State University, Museum of Natural Science, Baton Rouge, Louisiana 70893, USA.
http://www.lsu.edu/mns/

Monte L. Bean Life Science Museum

BYU: Monte L. Bean Life Science Museum, Brigham Young University, Provo, Utah 84602, USA.
https://mlbean.byu.edu/

Museum of Comparative Zoology, Harvard University

MCZ: Museum of Comparative Zoology, Harvard University, Cambridge, Massachusetts 02138, USA
http://www.mcz.harvard.edu/hmnh/index.html

National Museum of Natural History

USNM: National Museum of Natural History, Amphibians and Reptiles, Washington, D.C. 20560, USA.
https://naturalhistory.si.edu/

Natural History Museum of Los Angeles County

LACM: Natural History Museum of Los Angeles County,Herpetology, Los Angeles, California 90007, USA
Natural History Museum of Los Angeles County

Texas Natural History Collection

TNHC: Texas Memorial Museum, Texas Natural History Collection, 24th and Trinity, Austin, Texas 78705, USA.
https://integrativebio.utexas.edu/biodiversity-collections

The Canadian Museum of Nature

The Canadian Museum of Nature, Ottawa, Ontario, Canada.
http://nature.ca/en/home

The French National Museum of Natural History

MNHN: The French National Museum of Natural History, 57 Rue Cuvier, 75005 Paris, France
http://www.mnhn.fr/

The Museum of Vertebrate Zoology

MVZ: The Museum of Vertebrate Zoology, University of California, Berkeley, USA.
http://mvz.berkeley.edu/

The Natural History Museum in London

NHMUK: The Natural History Museum in London , Cromwell Rd, Kensington, London SW7 5BD, UK
http://www.nhm.ac.uk/

University of Colorado Museum

UCM: University of Colorado Museum, Herpetology, Campus Box 315, Boulder, Colorado 80309, USA.
http://www.colorado.edu/cumuseum/

University of Kansas Biodiversity Institute Herpetology Collection

KU: University of Kansas Biodiversity Institute, Herpetology , 345 Jayhawk Blvd., Lawrence, KS 66045
https://biodiversity.ku.edu/herpetology
