# Supplementary material for: Lowland extirpation of anuran populations on a tropical mountain
Source: PeerJ. 2017 Nov 15;5:e4059. doi: 10.7717/peerj.4059 (PMC5694215; doi:10.7717/peerj.4059)
Supplement: Table S3 [file peerj-05-4059-s004.pdf]

GBIF and Literature ID	LAT	LONG	Elevation (m)	*E_coqui*	*E_locustus*
686986656	18.30757	-65.77672	538	0	1
686986548	18.317617	-65.831095	463	0	1
686986568	18.296603	-65.841736	456	1	1
686986472	18.29872	-65.78565	674	1	1
886790124 , Gould et al 2008	18.269	-65.758	1010	0	1
1037809470	18.27221	-65.83351	985	1	1
887917349	18.29919	-65.78035	672	1	1
887170379	18.27557	-65.78866	687	0	1
686784538	18.303578	-65.777054	514	0	1
887917453	18.307633	-65.794455	1000	1	1
1037797851	18.29759	-65.78951	731	1	1
1037797855	18.301	-65.793	911	1	1
1037797847	18.300074	-65.793412	865	1	1
888426833, Drewry and Randy,1983	18.311	-65.791	1027	0	1
476566423	18.272	-65.829	1045	0	1
1037796685	18.269	-65.758	1011	0	1
686989627	18.29766	-65.78722	691	1	1
686992146	18.29053	-65.79074	683	0	0
686887253	18.306006	-65.774821	493	1	0
657377329	18.322304	-65.818666	344	1	0
543487714	18.29522	-65.78853	742	0	0
1037797516	18.321198	-65.81572	412	1	0
686989614	18.310852	-65.792862	1024	1	0
Joglar, 1998	18.30059444	-65.78538889	661	1	0
Joglar, 1998	18.30080556	-65.79319444	850	1	1
888144816	18.30674	-65.77775	560	1	0
1037796651	18.36415	-65.81964	39	0	0
886858926	18.329083	-65.820517	244	0	0
1037796213	18.34654	-65.7627	97	1	0
886890302	18.311866	-65.77722	489	0	0
1037797225	18.27055	-65.75945	985	0	0
1037797430	18.294862	-65.784617	758	1	0
686887247	18.303063	-65.78429	650	1	0
Drewry and Randy,1983	18.282739	-65.856976	600	1	1
866440579,Drewry and Randy,1983	18.32254	-65.817439	371	1	1
Gould et al 2008	18.303	-65.795	899	0	0
Gould et al 2008	18.296	-65.791	757	0	0
Gould et al 2008	18.292	-65.783	753	0	0
Gould et al 2008	18.283	-65.811	865	0	0
1037796671	18.332572	-65.783419	371	1	0
1037797443	18.322116	-65.82208	327	1	0
1321882617	18.3521	-65.766808	40	1	0
1037797764	18.241308	-65.7908	86	0	0
1321151764	18.3111	-65.7712	461	1	0
1319867316	18.3016	-65.7842	636	1	0
1037796479	18.343092	-65.761692	129	1	0

1037809453	18.322848	-65.765189	378	1	0
1037797448	18.28	-65.77	877	1	0
1037796689	18.34113	-65.82858	136	0	0
886858997	18.337257	-65.760628	213	0	0
686985846	18.334517	-65.825112	122	0	0

GBIF and Literature ID	LAT	LONG	Elevation (m)	*E_portoricensis*
686986656	18.30757	-65.77672	538	1
686986548	18.317617	-65.831095	463	0
686986568	18.296603	-65.841736	456	1
686986472	18.29872	-65.78565	674	1
886790124 , Gould et al 2008	18.269	-65.758	1010	1
1037809470	18.27221	-65.83351	985	1
887917349	18.29919	-65.78035	672	1
887170379	18.27557	-65.78866	687	0
686784538	18.303578	-65.777054	514	0
887917453	18.307633	-65.794455	1000	1
1037797851	18.29759	-65.78951	731	1
1037797855	18.301	-65.793	911	0
1037797847	18.300074	-65.793412	865	1
888426833, Drewry and Randy,1983	18.311	-65.791	1027	0
476566423	18.272	-65.829	1045	1
1037796685	18.269	-65.758	1011	0
686989627	18.29766	-65.78722	691	1
686992146	18.29053	-65.79074	683	0
686887253	18.306006	-65.774821	493	1
657377329	18.322304	-65.818666	344	1
543487714	18.29522	-65.78853	742	1
1037797516	18.321198	-65.81572	412	1
686989614	18.310852	-65.792862	1024	1
Joglar, 1998	18.30059444	-65.78538889	661	1
Joglar, 1998	18.30080556	-65.79319444	850	1
888144816	18.30674	-65.77775	560	0
1037796651	18.36415	-65.81964	39	0
886858926	18.329083	-65.820517	244	0
1037796213	18.34654	-65.7627	97	0
886890302	18.311866	-65.77722	489	0
1037797225	18.27055	-65.75945	985	1
1037797430	18.294862	-65.784617	758	1
686887247	18.303063	-65.78429	650	1
Drewry and Randy,1983	18.282739	-65.856976	600	1
866440579,Drewry and Randy,1983	18.32254	-65.817439	371	1
Gould et al 2008	18.303	-65.795	899	1
Gould et al 2008	18.296	-65.791	757	1
Gould et al 2008	18.292	-65.783	753	1
Gould et al 2008	18.283	-65.811	865	1
1037796671	18.332572	-65.783419	371	0
1037797443	18.322116	-65.82208	327	0
1321882617	18.3521	-65.766808	40	0
1037797764	18.241308	-65.7908	86	0
1321151764	18.3111	-65.7712	461	0
1319867316	18.3016	-65.7842	636	0
1037796479	18.343092	-65.761692	129	0
1037809453	18.322848	-65.765189	378	0
1037797448	18.28	-65.77	877	0
1037796689	18.34113	-65.82858	136	0
886858997	18.337257	-65.760628	213	0
686985846	18.334517	-65.825112	122	0

GBIF and Literature ID	LAT	LONG	Elevation (m)	*E_gryllus*
686986656	18.30757	-65.77672	538	0
686986548	18.317617	-65.831095	463	1
686986568	18.296603	-65.841736	456	1
686986472	18.29872	-65.78565	674	1
886790124 , Gould et al 2008	18.269	-65.758	1010	0
1037809470	18.27221	-65.83351	985	0
887917349	18.29919	-65.78035	672	0
887170379	18.27557	-65.78866	687	0
686784538	18.303578	-65.777054	514	0
887917453	18.307633	-65.794455	1000	0
1037797851	18.29759	-65.78951	731	0
1037797855	18.301	-65.793	911	0
1037797847	18.300074	-65.793412	865	1
888426833, Drewry and Randy,1983	18.311	-65.791	1027	1
476566423	18.272	-65.829	1045	0
1037796685	18.269	-65.758	1011	0
686989627	18.29766	-65.78722	691	1
686992146	18.29053	-65.79074	683	0
686887253	18.306006	-65.774821	493	0
657377329	18.322304	-65.818666	344	0
543487714	18.29522	-65.78853	742	1
1037797516	18.321198	-65.81572	412	0
686989614	18.310852	-65.792862	1024	1
Joglar, 1998	18.30059444	-65.78538889	661	0
Joglar, 1998	18.30080556	-65.79319444	850	0
888144816	18.30674	-65.77775	560	0
1037796651	18.36415	-65.81964	39	0
886858926	18.329083	-65.820517	244	0
1037796213	18.34654	-65.7627	97	0
886890302	18.311866	-65.77722	489	0
1037797225	18.27055	-65.75945	985	0
1037797430	18.294862	-65.784617	758	0
686887247	18.303063	-65.78429	650	0
Drewry and Randy,1983	18.282739	-65.856976	600	1
866440579,Drewry and Randy,1983	18.32254	-65.817439	371	1
Gould et al 2008	18.303	-65.795	899	0
Gould et al 2008	18.296	-65.791	757	0
Gould et al 2008	18.292	-65.783	753	1
Gould et al 2008	18.283	-65.811	865	0
1037796671	18.332572	-65.783419	371	0
1037797443	18.322116	-65.82208	327	0
1321882617	18.3521	-65.766808	40	0
1037797764	18.241308	-65.7908	86	0
1321151764	18.3111	-65.7712	461	0
1319867316	18.3016	-65.7842	636	0
1037796479	18.343092	-65.761692	129	0

1037809453	18.322848	-65.765189	378	0
1037797448	18.28	-65.77	877	0
1037796689	18.34113	-65.82858	136	0
886858997	18.337257	-65.760628	213	0
686985846	18.334517	-65.825112	122	0

GBIF and Literature ID	LAT	LONG	Elevation (m)	*E_brittoni*
686986656	18.30757	-65.77672	538	1
686986548	18.317617	-65.831095	463	0
686986568	18.296603	-65.841736	456	1
686986472	18.29872	-65.78565	674	0
886790124 , Gould et al 2008	18.269	-65.758	1010	0
1037809470	18.27221	-65.83351	985	0
887917349	18.29919	-65.78035	672	0
887170379	18.27557	-65.78866	687	0
686784538	18.303578	-65.777054	514	0
887917453	18.307633	-65.794455	1000	0
1037797851	18.29759	-65.78951	731	0
1037797855	18.301	-65.793	911	0
1037797847	18.300074	-65.793412	865	0
888426833, Drewry and Randy,1983	18.311	-65.791	1027	0
476566423	18.272	-65.829	1045	0
1037796685	18.269	-65.758	1011	0
686989627	18.29766	-65.78722	691	0
686992146	18.29053	-65.79074	683	0
686887253	18.306006	-65.774821	493	0
657377329	18.322304	-65.818666	344	0
543487714	18.29522	-65.78853	742	0
1037797516	18.321198	-65.81572	412	0
686989614	18.310852	-65.792862	1024	0
Joglar, 1998	18.30059444	-65.78538889	661	0
Joglar, 1998	18.30080556	-65.79319444	850	0
888144816	18.30674	-65.77775	560	1
1037796651	18.36415	-65.81964	39	1
886858926	18.329083	-65.820517	244	1
1037796213	18.34654	-65.7627	97	1
886890302	18.311866	-65.77722	489	0
1037797225	18.27055	-65.75945	985	0
1037797430	18.294862	-65.784617	758	1
686887247	18.303063	-65.78429	650	0
Drewry and Randy,1983	18.282739	-65.856976	600	1
866440579,Drewry and Randy,1983	18.32254	-65.817439	371	1
Gould et al 2008	18.303	-65.795	899	0
Gould et al 2008	18.296	-65.791	757	0
Gould et al 2008	18.292	-65.783	753	0
Gould et al 2008	18.283	-65.811	865	0
1037796671	18.332572	-65.783419	371	0
1037797443	18.322116	-65.82208	327	0
1321882617	18.3521	-65.766808	40	0
1037797764	18.241308	-65.7908	86	0
1321151764	18.3111	-65.7712	461	0
1319867316	18.3016	-65.7842	636	0
1037796479	18.343092	-65.761692	129	0
1037809453	18.322848	-65.765189	378	0
1037797448	18.28	-65.77	877	0
1037796689	18.34113	-65.82858	136	0
886858997	18.337257	-65.760628	213	1
686985846	18.334517	-65.825112	122	0

GBIF and Literature ID	LAT	LONG	Elevation (m)	*E_hedricki*
686986656	18.30757	-65.77672	538	0
686986548	18.317617	-65.831095	463	0
686986568	18.296603	-65.841736	456	0
686986472	18.29872	-65.78565	674	0
886790124 , Gould et al 2008	18.269	-65.758	1010	0
1037809470	18.27221	-65.83351	985	0
887917349	18.29919	-65.78035	672	0
887170379	18.27557	-65.78866	687	0
686784538	18.303578	-65.777054	514	0
887917453	18.307633	-65.794455	1000	0
1037797851	18.29759	-65.78951	731	0
1037797855	18.301	-65.793	911	0
1037797847	18.300074	-65.793412	865	0
888426833, Drewry and Randy,1983	18.311	-65.791	1027	0
476566423	18.272	-65.829	1045	0
1037796685	18.269	-65.758	1011	0
686989627	18.29766	-65.78722	691	0
686992146	18.29053	-65.79074	683	0
686887253	18.306006	-65.774821	493	0
657377329	18.322304	-65.818666	344	0
543487714	18.29522	-65.78853	742	0
1037797516	18.321198	-65.81572	412	1
686989614	18.310852	-65.792862	1024	0
Joglar, 1998	18.30059444	-65.78538889	661	1
Joglar, 1998	18.30080556	-65.79319444	850	0
888144816	18.30674	-65.77775	560	0
1037796651	18.36415	-65.81964	39	0
886858926	18.329083	-65.820517	244	0
1037796213	18.34654	-65.7627	97	0
886890302	18.311866	-65.77722	489	0
1037797225	18.27055	-65.75945	985	0
1037797430	18.294862	-65.784617	758	0
686887247	18.303063	-65.78429	650	0
Drewry and Randy,1983	18.282739	-65.856976	600	1
866440579,Drewry and Randy,1983	18.32254	-65.817439	371	1
Gould et al 2008	18.303	-65.795	899	0
Gould et al 2008	18.296	-65.791	757	0
Gould et al 2008	18.292	-65.783	753	0
Gould et al 2008	18.283	-65.811	865	0
1037796671	18.332572	-65.783419	371	0
1037797443	18.322116	-65.82208	327	0
1321882617	18.3521	-65.766808	40	0
1037797764	18.241308	-65.7908	86	0
1321151764	18.3111	-65.7712	461	0
1319867316	18.3016	-65.7842	636	0
1037796479	18.343092	-65.761692	129	0
1037809453	18.322848	-65.765189	378	0
1037797448	18.28	-65.77	877	0
1037796689	18.34113	-65.82858	136	0
886858997	18.337257	-65.760628	213	0
686985846	18.334517	-65.825112	122	0

GBIF and Literature ID	LAT	LONG	Elevation (m)	*E_richmondi*
686986656	18.30757	-65.77672	538	1
686986548	18.317617	-65.831095	463	0
686986568	18.296603	-65.841736	456	1
686986472	18.29872	-65.78565	674	1
886790124 , Gould et al 2008	18.269	-65.758	1010	0
1037809470	18.27221	-65.83351	985	0
887917349	18.29919	-65.78035	672	1
887170379	18.27557	-65.78866	687	0
686784538	18.303578	-65.777054	514	0
887917453	18.307633	-65.794455	1000	0
1037797851	18.29759	-65.78951	731	0
1037797855	18.301	-65.793	911	1
1037797847	18.300074	-65.793412	865	1
888426833, Drewry and Randy,1983	18.311	-65.791	1027	1
476566423	18.272	-65.829	1045	0
1037796685	18.269	-65.758	1011	0
686989627	18.29766	-65.78722	691	1
686992146	18.29053	-65.79074	683	0
686887253	18.306006	-65.774821	493	0
657377329	18.322304	-65.818666	344	1
543487714	18.29522	-65.78853	742	1
1037797516	18.321198	-65.81572	412	0
686989614	18.310852	-65.792862	1024	1
Joglar, 1998	18.30059444	-65.78538889	661	1
Joglar, 1998	18.30080556	-65.79319444	850	1
888144816	18.30674	-65.77775	560	0
1037796651	18.36415	-65.81964	39	0
886858926	18.329083	-65.820517	244	0
1037796213	18.34654	-65.7627	97	0
886890302	18.311866	-65.77722	489	1
1037797225	18.27055	-65.75945	985	0
1037797430	18.294862	-65.784617	758	0
686887247	18.303063	-65.78429	650	0
Drewry and Randy,1983	18.282739	-65.856976	600	1
866440579,Drewry and Randy,1983	18.32254	-65.817439	371	1
Gould et al 2008	18.303	-65.795	899	0
Gould et al 2008	18.296	-65.791	757	0
Gould et al 2008	18.292	-65.783	753	0
Gould et al 2008	18.283	-65.811	865	0
1037796671	18.332572	-65.783419	371	0
1037797443	18.322116	-65.82208	327	0
1321882617	18.3521	-65.766808	40	0
1037797764	18.241308	-65.7908	86	0
1321151764	18.3111	-65.7712	461	0
1319867316	18.3016	-65.7842	636	0
1037796479	18.343092	-65.761692	129	0
1037809453	18.322848	-65.765189	378	0
1037797448	18.28	-65.77	877	0
1037796689	18.34113	-65.82858	136	0
886858997	18.337257	-65.760628	213	0
686985846	18.334517	-65.825112	122	0

GBIF and Literature ID	LAT	LONG	Elevation (m)	*E_wightmanae*
686986656	18.30757	-65.77672	538	1
686986548	18.317617	-65.831095	463	0
686986568	18.296603	-65.841736	456	1
686986472	18.29872	-65.78565	674	1
886790124 , Gould et al 2008	18.269	-65.758	1010	0
1037809470	18.27221	-65.83351	985	1
887917349	18.29919	-65.78035	672	1
887170379	18.27557	-65.78866	687	0
686784538	18.303578	-65.777054	514	1
887917453	18.307633	-65.794455	1000	1
1037797851	18.29759	-65.78951	731	0
1037797855	18.301	-65.793	911	0
1037797847	18.300074	-65.793412	865	1
888426833, Drewry and Randy,1983	18.311	-65.791	1027	0
476566423	18.272	-65.829	1045	0
1037796685	18.269	-65.758	1011	0
686989627	18.29766	-65.78722	691	1
686992146	18.29053	-65.79074	683	1
686887253	18.306006	-65.774821	493	1
657377329	18.322304	-65.818666	344	1
543487714	18.29522	-65.78853	742	1
1037797516	18.321198	-65.81572	412	1
686989614	18.310852	-65.792862	1024	0
Joglar, 1998	18.30059444	-65.78538889	661	1
Joglar, 1998	18.30080556	-65.79319444	850	1
888144816	18.30674	-65.77775	560	0
1037796651	18.36415	-65.81964	39	0
886858926	18.329083	-65.820517	244	0
1037796213	18.34654	-65.7627	97	0
886890302	18.311866	-65.77722	489	0
1037797225	18.27055	-65.75945	985	0
1037797430	18.294862	-65.784617	758	0
686887247	18.303063	-65.78429	650	0
Drewry and Randy,1983	18.282739	-65.856976	600	1
866440579,Drewry and Randy,1983	18.32254	-65.817439	371	1
Gould et al 2008	18.303	-65.795	899	0
Gould et al 2008	18.296	-65.791	757	0
Gould et al 2008	18.292	-65.783	753	0
Gould et al 2008	18.283	-65.811	865	0
1037796671	18.332572	-65.783419	371	0
1037797443	18.322116	-65.82208	327	0
1321882617	18.3521	-65.766808	40	0
1037797764	18.241308	-65.7908	86	0
1321151764	18.3111	-65.7712	461	0
1319867316	18.3016	-65.7842	636	0
1037796479	18.343092	-65.761692	129	0
1037809453	18.322848	-65.765189	378	0
1037797448	18.28	-65.77	877	0
1037796689	18.34113	-65.82858	136	0
886858997	18.337257	-65.760628	213	0
686985846	18.334517	-65.825112	122	0

GBIF and Literature ID	LAT	LONG	Elevation (m)	*E_unicolor*
686986656	18.30757	-65.77672	538	0
686986548	18.317617	-65.831095	463	0
686986568	18.296603	-65.841736	456	0
686986472	18.29872	-65.78565	674	0
886790124 , Gould et al 2008	18.269	-65.758	1010	0
1037809470	18.27221	-65.83351	985	0
887917349	18.29919	-65.78035	672	0
887170379	18.27557	-65.78866	687	0
686784538	18.303578	-65.777054	514	0
887917453	18.307633	-65.794455	1000	0
1037797851	18.29759	-65.78951	731	0
1037797855	18.301	-65.793	911	0
1037797847	18.300074	-65.793412	865	0
888426833, Drewry and Randy,1983	18.311	-65.791	1027	1
476566423	18.272	-65.829	1045	0
1037796685	18.269	-65.758	1011	0
686989627	18.29766	-65.78722	691	0
686992146	18.29053	-65.79074	683	0
686887253	18.306006	-65.774821	493	0
657377329	18.322304	-65.818666	344	0
543487714	18.29522	-65.78853	742	0
1037797516	18.321198	-65.81572	412	0
686989614	18.310852	-65.792862	1024	1
Joglar, 1998	18.30059444	-65.78538889	661	0
Joglar, 1998	18.30080556	-65.79319444	850	1
888144816	18.30674	-65.77775	560	0
1037796651	18.36415	-65.81964	39	0
886858926	18.329083	-65.820517	244	0
1037796213	18.34654	-65.7627	97	0
886890302	18.311866	-65.77722	489	0
1037797225	18.27055	-65.75945	985	0
1037797430	18.294862	-65.784617	758	0
686887247	18.303063	-65.78429	650	0
Drewry and Randy,1983	18.282739	-65.856976	600	0
866440579,Drewry and Randy,1983	18.32254	-65.817439	371	0
Gould et al 2008	18.303	-65.795	899	0
Gould et al 2008	18.296	-65.791	757	0
Gould et al 2008	18.292	-65.783	753	0
Gould et al 2008	18.283	-65.811	865	0
1037796671	18.332572	-65.783419	371	0
1037797443	18.322116	-65.82208	327	0
1321882617	18.3521	-65.766808	40	0
1037797764	18.241308	-65.7908	86	0
1321151764	18.3111	-65.7712	461	0
1319867316	18.3016	-65.7842	636	0
1037796479	18.343092	-65.761692	129	0
1037809453	18.322848	-65.765189	378	0
1037797448	18.28	-65.77	877	0
1037796689	18.34113	-65.82858	136	0
886858997	18.337257	-65.760628	213	0
686985846	18.334517	-65.825112	122	0

GBIF and Literature ID	LAT	LONG	Elevation (m)	*E_antillensis*
686986656	18.30757	-65.77672	538	0
686986548	18.317617	-65.831095	463	0
686986568	18.296603	-65.841736	456	1
686986472	18.29872	-65.78565	674	0
886790124 , Gould et al 2008	18.269	-65.758	1010	0
1037809470	18.27221	-65.83351	985	0
887917349	18.29919	-65.78035	672	0
887170379	18.27557	-65.78866	687	0
686784538	18.303578	-65.777054	514	0
887917453	18.307633	-65.794455	1000	0
1037797851	18.29759	-65.78951	731	1
1037797855	18.301	-65.793	911	0
1037797847	18.300074	-65.793412	865	0
888426833, Drewry and Randy,1983	18.311	-65.791	1027	0
476566423	18.272	-65.829	1045	0
1037796685	18.269	-65.758	1011	0
686989627	18.29766	-65.78722	691	0
686992146	18.29053	-65.79074	683	0
686887253	18.306006	-65.774821	493	0
657377329	18.322304	-65.818666	344	0
543487714	18.29522	-65.78853	742	0
1037797516	18.321198	-65.81572	412	1
686989614	18.310852	-65.792862	1024	0
Joglar, 1998	18.30059444	-65.78538889	661	0
Joglar, 1998	18.30080556	-65.79319444	850	0
888144816	18.30674	-65.77775	560	0
1037796651	18.36415	-65.81964	39	1
886858926	18.329083	-65.820517	244	0
1037796213	18.34654	-65.7627	97	0
886890302	18.311866	-65.77722	489	0
1037797225	18.27055	-65.75945	985	0
1037797430	18.294862	-65.784617	758	0
686887247	18.303063	-65.78429	650	0
Drewry and Randy,1983	18.282739	-65.856976	600	0
866440579,Drewry and Randy,1983	18.32254	-65.817439	371	1
Gould et al 2008	18.303	-65.795	899	0
Gould et al 2008	18.296	-65.791	757	0
Gould et al 2008	18.292	-65.783	753	0
Gould et al 2008	18.283	-65.811	865	0
1037796671	18.332572	-65.783419	371	1
1037797443	18.322116	-65.82208	327	1
1321882617	18.3521	-65.766808	40	1
1037797764	18.241308	-65.7908	86	1
1321151764	18.3111	-65.7712	461	0
1319867316	18.3016	-65.7842	636	0
1037796479	18.343092	-65.761692	129	0
1037809453	18.322848	-65.765189	378	0
1037797448	18.28	-65.77	877	0
1037796689	18.34113	-65.82858	136	1
886858997	18.337257	-65.760628	213	0
686985846	18.334517	-65.825112	122	0

GBIF and Literature ID	LAT	LONG	Elevation (m)	*E_cochranae*
686986656	18.30757	-65.77672	538	0
686986548	18.317617	-65.831095	463	0
686986568	18.296603	-65.841736	456	0
686986472	18.29872	-65.78565	674	0
886790124 , Gould et al 2008	18.269	-65.758	1010	0
1037809470	18.27221	-65.83351	985	0
887917349	18.29919	-65.78035	672	0
887170379	18.27557	-65.78866	687	0
686784538	18.303578	-65.777054	514	0
887917453	18.307633	-65.794455	1000	0
1037797851	18.29759	-65.78951	731	0
1037797855	18.301	-65.793	911	0
1037797847	18.300074	-65.793412	865	0
888426833, Drewry and Randy,1983	18.311	-65.791	1027	0
476566423	18.272	-65.829	1045	0
1037796685	18.269	-65.758	1011	0
686989627	18.29766	-65.78722	691	0
686992146	18.29053	-65.79074	683	0
686887253	18.306006	-65.774821	493	0
657377329	18.322304	-65.818666	344	0
543487714	18.29522	-65.78853	742	1
1037797516	18.321198	-65.81572	412	0
686989614	18.310852	-65.792862	1024	0
Joglar, 1998	18.30059444	-65.78538889	661	0
Joglar, 1998	18.30080556	-65.79319444	850	0
888144816	18.30674	-65.77775	560	0
1037796651	18.36415	-65.81964	39	0
886858926	18.329083	-65.820517	244	0
1037796213	18.34654	-65.7627	97	1
886890302	18.311866	-65.77722	489	0
1037797225	18.27055	-65.75945	985	0
1037797430	18.294862	-65.784617	758	0
686887247	18.303063	-65.78429	650	0
Drewry and Randy,1983	18.282739	-65.856976	600	0
866440579,Drewry and Randy,1983	18.32254	-65.817439	371	0
Gould et al 2008	18.303	-65.795	899	0
Gould et al 2008	18.296	-65.791	757	0
Gould et al 2008	18.292	-65.783	753	0
Gould et al 2008	18.283	-65.811	865	0
1037796671	18.332572	-65.783419	371	0
1037797443	18.322116	-65.82208	327	0
1321882617	18.3521	-65.766808	40	1
1037797764	18.241308	-65.7908	86	0
1321151764	18.3111	-65.7712	461	0
1319867316	18.3016	-65.7842	636	0
1037796479	18.343092	-65.761692	129	0
1037809453	18.322848	-65.765189	378	0
1037797448	18.28	-65.77	877	0
1037796689	18.34113	-65.82858	136	0
886858997	18.337257	-65.760628	213	0
686985846	18.334517	-65.825112	122	0

GBIF and Literature ID	LAT	LONG	Elevation (m)	*L_albilabris*
686986656	18.30757	-65.77672	538	0
686986548	18.317617	-65.831095	463	1
686986568	18.296603	-65.841736	456	0
686986472	18.29872	-65.78565	674	1
886790124 , Gould et al 2008	18.269	-65.758	1010	1
1037809470	18.27221	-65.83351	985	0
887917349	18.29919	-65.78035	672	0
887170379	18.27557	-65.78866	687	0
686784538	18.303578	-65.777054	514	0
887917453	18.307633	-65.794455	1000	0
1037797851	18.29759	-65.78951	731	0
1037797855	18.301	-65.793	911	0
1037797847	18.300074	-65.793412	865	0
888426833, Drewry and Randy,1983	18.311	-65.791	1027	0
476566423	18.272	-65.829	1045	0
1037796685	18.269	-65.758	1011	0
686989627	18.29766	-65.78722	691	1
686992146	18.29053	-65.79074	683	1
686887253	18.306006	-65.774821	493	0
657377329	18.322304	-65.818666	344	1
543487714	18.29522	-65.78853	742	0
1037797516	18.321198	-65.81572	412	1
686989614	18.310852	-65.792862	1024	0
Joglar, 1998	18.30059444	-65.78538889	661	0
Joglar, 1998	18.30080556	-65.79319444	850	0
888144816	18.30674	-65.77775	560	0
1037796651	18.36415	-65.81964	39	0
886858926	18.329083	-65.820517	244	0
1037796213	18.34654	-65.7627	97	1
886890302	18.311866	-65.77722	489	0
1037797225	18.27055	-65.75945	985	0
1037797430	18.294862	-65.784617	758	0
686887247	18.303063	-65.78429	650	0
Drewry and Randy,1983	18.282739	-65.856976	600	0
866440579,Drewry and Randy,1983	18.32254	-65.817439	371	0
Gould et al 2008	18.303	-65.795	899	0
Gould et al 2008	18.296	-65.791	757	0
Gould et al 2008	18.292	-65.783	753	0
Gould et al 2008	18.283	-65.811	865	0
1037796671	18.332572	-65.783419	371	0
1037797443	18.322116	-65.82208	327	0
1321882617	18.3521	-65.766808	40	0
1037797764	18.241308	-65.7908	86	0
1321151764	18.3111	-65.7712	461	0
1319867316	18.3016	-65.7842	636	0
1037796479	18.343092	-65.761692	129	0
1037809453	18.322848	-65.765189	378	0
1037797448	18.28	-65.77	877	0
1037796689	18.34113	-65.82858	136	0
886858997	18.337257	-65.760628	213	0
686985846	18.334517	-65.825112	122	0

GBIF and Literature ID	LAT	LONG	Elevation (m)	*E_eneidae*
686986656	18.30757	-65.77672	538	1
686986548	18.317617	-65.831095	463	0
686986568	18.296603	-65.841736	456	0
686986472	18.29872	-65.78565	674	1
886790124 , Gould et al 2008	18.269	-65.758	1010	0
1037809470	18.27221	-65.83351	985	0
887917349	18.29919	-65.78035	672	0
887170379	18.27557	-65.78866	687	0
686784538	18.303578	-65.777054	514	1
887917453	18.307633	-65.794455	1000	1
1037797851	18.29759	-65.78951	731	0
1037797855	18.301	-65.793	911	0
1037797847	18.300074	-65.793412	865	1
888426833, Drewry and Randy,1983	18.311	-65.791	1027	0
476566423	18.272	-65.829	1045	0
1037796685	18.269	-65.758	1011	0
686989627	18.29766	-65.78722	691	1
686992146	18.29053	-65.79074	683	0
686887253	18.306006	-65.774821	493	0
657377329	18.322304	-65.818666	344	0
543487714	18.29522	-65.78853	742	1
1037797516	18.321198	-65.81572	412	0
686989614	18.310852	-65.792862	1024	0
Joglar, 1998	18.30059444	-65.78538889	661	0
Joglar, 1998	18.30080556	-65.79319444	850	0
888144816	18.30674	-65.77775	560	0
1037796651	18.36415	-65.81964	39	0
886858926	18.329083	-65.820517	244	0
1037796213	18.34654	-65.7627	97	0
886890302	18.311866	-65.77722	489	0
1037797225	18.27055	-65.75945	985	0
1037797430	18.294862	-65.784617	758	0
686887247	18.303063	-65.78429	650	0
Drewry and Randy,1983	18.282739	-65.856976	600	1
866440579,Drewry and Randy,1983	18.32254	-65.817439	371	1
Gould et al 2008	18.303	-65.795	899	0
Gould et al 2008	18.296	-65.791	757	0
Gould et al 2008	18.292	-65.783	753	0
Gould et al 2008	18.283	-65.811	865	0
1037796671	18.332572	-65.783419	371	0
1037797443	18.322116	-65.82208	327	0
1321882617	18.3521	-65.766808	40	0
1037797764	18.241308	-65.7908	86	0
1321151764	18.3111	-65.7712	461	0
1319867316	18.3016	-65.7842	636	0
1037796479	18.343092	-65.761692	129	0
1037809453	18.322848	-65.765189	378	0
1037797448	18.28	-65.77	877	0
1037796689	18.34113	-65.82858	136	0
886858997	18.337257	-65.760628	213	0
686985846	18.334517	-65.825112	122	0

GBIF and Literature ID	LAT	LONG	Elevation (m)	*E_karlschmidti*
686986656	18.30757	-65.77672	538	1
686986548	18.317617	-65.831095	463	0
686986568	18.296603	-65.841736	456	0
686986472	18.29872	-65.78565	674	1
886790124 , Gould et al 2008	18.269	-65.758	1010	0
1037809470	18.27221	-65.83351	985	0
887917349	18.29919	-65.78035	672	0
887170379	18.27557	-65.78866	687	0
686784538	18.303578	-65.777054	514	1
887917453	18.307633	-65.794455	1000	0
1037797851	18.29759	-65.78951	731	0
1037797855	18.301	-65.793	911	0
1037797847	18.300074	-65.793412	865	0
888426833, Drewry and Randy,1983	18.311	-65.791	1027	0
476566423	18.272	-65.829	1045	0
1037796685	18.269	-65.758	1011	0
686989627	18.29766	-65.78722	691	1
686992146	18.29053	-65.79074	683	0
686887253	18.306006	-65.774821	493	0
657377329	18.322304	-65.818666	344	0
543487714	18.29522	-65.78853	742	0
1037797516	18.321198	-65.81572	412	0
686989614	18.310852	-65.792862	1024	0
Joglar, 1998	18.30059444	-65.78538889	661	0
Joglar, 1998	18.30080556	-65.79319444	850	0
888144816	18.30674	-65.77775	560	0
1037796651	18.36415	-65.81964	39	0
886858926	18.329083	-65.820517	244	0
1037796213	18.34654	-65.7627	97	0
886890302	18.311866	-65.77722	489	0
1037797225	18.27055	-65.75945	985	0
1037797430	18.294862	-65.784617	758	0
686887247	18.303063	-65.78429	650	0
Drewry and Randy,1983	18.282739	-65.856976	600	1
866440579,Drewry and Randy,1983	18.32254	-65.817439	371	1
Gould et al 2008	18.303	-65.795	899	0
Gould et al 2008	18.296	-65.791	757	0
Gould et al 2008	18.292	-65.783	753	0
Gould et al 2008	18.283	-65.811	865	0
1037796671	18.332572	-65.783419	371	0
1037797443	18.322116	-65.82208	327	0
1321882617	18.3521	-65.766808	40	0
1037797764	18.241308	-65.7908	86	0
1321151764	18.3111	-65.7712	461	0
1319867316	18.3016	-65.7842	636	0
1037796479	18.343092	-65.761692	129	0
1037809453	18.322848	-65.765189	378	0
1037797448	18.28	-65.77	877	0
1037796689	18.34113	-65.82858	136	0
886858997	18.337257	-65.760628	213	0
686985846	18.334517	-65.825112	122	0
