# Supplementary material for: Lowland extirpation of anuran populations on a tropical mountain
Source: PeerJ. 2017 Nov 15;5:e4059. doi: 10.7717/peerj.4059 (PMC5694215; doi:10.7717/peerj.4059)
Supplement: Table S4 [file peerj-05-4059-s005.docx]

Table S4. Nine alternative occupancy models for the current distribution data sets.

| **Models** | **Parameters/ Covariates** | |
| --- | --- | --- |
|  | **Occupancy** | **Detection** |
| 1 | (.) | (.) |
| 2 | (.) | Elevation |
| 3 | (.) | Elevation+Elevation^2^ |
| 4 | Elevation | (.) |
| 5 | Elevation | Elevation |
| 6 | Elevation | Elevation+Elevation^2^ |
| 7 | Elevation+Elevation^2^ | (.) |
| 8 | Elevation+Elevation^2^ | Elevation |
| 9 | Elavation+Elevation^2^ | Elevation+Elevation^2^ |
