# Supplementary material for: Lowland extirpation of anuran populations on a tropical mountain
Source: PeerJ. 2017 Nov 15;5:e4059. doi: 10.7717/peerj.4059 (PMC5694215; doi:10.7717/peerj.4059)
Supplement: Table S5 [file peerj-05-4059-s006.docx]

Table S5. Cumulative AIC weights based on 9 occupancy models averaged using the AIC weights.

| **Species** | ***ψ*(.)** | ***ψ*(elev)** | ***ψ*(elev+elev^2^)** | **Average *ψ*** |
| --- | --- | --- | --- | --- |
| E. *portoricensis* | 0.00 | 0.50 | 0.50 | 0.428678332 |
| *E. gryllus* | 0.00 | 0.65 | 0.35 | 0.167315985 |
| *E. locustus* | 0.55 | 0.35 | 0.10 | 0.112006105 |
| *E. richmondi* | 0.06 | 0.09 | 0.85 | 0.041255564 |
| *E. wightmanae* | 0.60 | 0.25 | 0.15 | 0.148373739 |
| *E. hedricki* | 0.00 | 0.00 | 1.00 | 0.361879673 |
| *E. unicolor* | 0.00 | 0.54 | 0.45 | 0.411544065 |
| *E. brittoni* | 0.00 | 0.13 | 0.87 | 0.468129876 |
| *L. albilabris* | 0.33 | 0.49 | 0.18 | 0.272796096 |
| *E. coqui* | NA | NA | NA | NA |
| *E. antillensis* | 0.01 | 0.70 | 0.29 | 0.048417482 |
| *E. cochranae* | 0.18 | 0.55 | 0.27 | 0.035736117 |
| *E. eneidae* | NA | NA | NA | NA |
| *E. karlschmidti* | NA | NA | NA | NA |
|  |  |  |  |  |
| **Species** | ***p*(.)** | ***p*(elev)** | ***p*(elev+elev^2^)** | **Average *p*** |
| E. portoricensis | 0.00 | 0.00 | 1.00 | 0.983161721 |
| *E. gryllus* | 0.00 | 0.00 | 1.00 | 0.871616472 |
| *E. locustus* | 0.00 | 0.90 | 0.11 | 0.404871014 |
| *E. richmondi* | 0.61 | 0.27 | 0.12 | 0.999982 |
| *E. wightmanae* | 0.45 | 0.20 | 0.35 | 0.85035349 |
| *E. hedricki* | 0.00 | 0.53 | 0.47 | 0.671874282 |
| *E. unicolor* | 0.00 | 0.58 | 0.41 | 0.57750989 |
| *E. brittoni* | 0.62 | 0.27 | 0.11 | 0.801602222 |
| *L. albilabris* | 0.00 | 0.00 | 1.00 | 0.453456274 |
| *E. coqui* | NA | NA | NA | NA |
| *E. antillensis* | 0.48 | 0.35 | 0.17 | 0.715446727 |
| *E. cochranae* | 0.60 | 0.32 | 0.08 | 0.946178075 |
| *E. eneidae* | NA | NA | NA | NA |
| *E. karlschmidti* | NA | NA | NA | NA |
