# Supplementary material for: Lowland extirpation of anuran populations on a tropical mountain
Source: PeerJ. 2017 Nov 15;5:e4059. doi: 10.7717/peerj.4059 (PMC5694215; doi:10.7717/peerj.4059)
Supplement: Table S6 — Given are the number of parameters (#Par); the log-likelihhod (logLik); twice the negative log-likelihhod (AIC); the relative difference in AIC values compared to the top-ranked model (ΔAIC) and the AIC model weights (AICwt). [file peerj-05-4059-s007.docx]

Table S6. Summary of GLM model selection procedure for the historical dataset of 14 species of native frogs in the Luquillo Mountains, Puerto Rico. Given are the number of parameters (k); twice the negative log-likelihhod (-2loglike); Akaike Information Criteria (AIC); the relative difference in AIC values compared to the top-ranked model (∆AIC) and the AIC model weights (AICwt)

| **Model** | **K** | **-2logLike** | **AIC** | **∆AIC** | **AICwt** |
| --- | --- | --- | --- | --- | --- |
| ***E. brittoni*** |  |  |  |  |  |
| Occupancy(elevation) | 2 | 44.282 | 48.5 | 0 | 0.516 |
| Occupancy(elevation^2^) | 3 | 42.46 | 49 | 0.44 | 0.415 |
| Occupancy(.) | 1 | 50.482 | 52.6 | 4.03 | 0.069 |
| ***E. locustus*** |  |  |  |  |  |
| Occupancy(elevation) | 2 | 58.28 | 62.5 | 0 | 0.67 |
| Occupancy(elevation^2^) | 3 | 57.52 | 64 | 1.5 | 0.31 |
| Occupancy(.) | 1 | 67.35 | 69.4 | 6.9 | 0.02 |
| ***E. gryllus*** |  |  |  |  |  |
| Occupancy(.) | 1 | 53.182 | 55.3 | 0 | 0.378 |
| Occupancy(elevation^2^) | 3 | 49.102 | 55.6 | 0.35 | 0.318 |
| Occupancy(elevation) | 2 | 51.45 | 55.7 | 0.44 | 0.304 |
| ***E. richmondi*** |  |  |  |  |  |
| Occupancy(elevation^2^) | 3 | 55.992 | 62.5 | 0 | 0.54 |
| Occupancy(.) | 1 | 61.792 | 63.9 | 1.37 | 0.27 |
| Occupancy(elevation) | 2 | 60.35 | 64.6 | 2.1 | 0.19 |
| ***E. wightmanae*** |  |  |  |  |  |
| Occupancy(elevation^2^) | 3 | 54.666 | 61.2 | 0 | 0.93 |
| Occupancy(.) | 1 | 64.924 | 67 | 5.83 | 0.05 |
| Occupancy(elevation) | 2 | 64.572 | 68.8 | 7.64 | 0.02 |
| ***E. portoricensis*** |  |  |  |  |  |
| Occupancy(elevation^2^) | 3 | 52.528 | 59 | 0 | 0.704 |
| Occupancy(elevation) | 2 | 56.528 | 60.8 | 1.74 | 0.295 |
| Occupancy(.) | 1 | 70.524 | 72.6 | 13.57 | 0.001 |
| ***E. unicolor*** |  |  |  |  |  |
| Occupancy(elevation) | 2 | 15.348 | 19.6 | 0 | 0.71 |
| Occupancy(elevation^2^) | 3 | 15.234 | 21.7 | 2.15 | 0.24 |
| Occupancy(.) | 1 | 22.82 | 24.9 | 5.3 | 0.05 |
| ***E. hedricki*** |  |  |  |  |  |
| Occupancy(elevation^2^) | 3 | 22.218 | 28.7 | 0 | 0.589 |
| Occupancy(.) | 1 | 28.042 | 30.1 | 1.39 | 0.293 |
| Occupancy(elevation) | 2 | 27.7 | 32 | 3.22 | 0.118 |

| **Model** | **k** | **-2logLike** | **AIC** | **∆AIC** | **AICwt** |
| --- | --- | --- | --- | --- | --- |
| ***E. antillensis*** |  |  |  |  |  |
| Occupancy(elevation) | 2 | 36.864 | 41.1 | 0 | 0.709 |
| Occupancy(elevation^2^) | 3 | 36.402 | 42.9 | 1.8 | 0.289 |
| Occupancy(.) | 1 | 50.482 | 52.6 | 11.45 | 0.002 |
| ***E. cochranae*** |  |  |  |  |  |
| Occupancy(elevation) | 2 | 19.436 | 23.7 | 0 | 0.477 |
| Occupancy(elevation^2^) | 3 | 18.366 | 24.9 | 1.19 | 0.263 |
| Occupancy(.) | 1 | 22.82 | 24.9 | 1.22 | 0.26 |
| ***L. albilabris*** |  |  |  |  |  |
| Occupancy(.) | 1 | 44.312 | 46.4 | 0 | 0.642 |
| Occupancy(elevation) | 2 | 44.066 | 48.3 | 1.92 | 0.246 |
| Occupancy(elevation^2^) | 3 | 43.368 | 49.9 | 3.49 | 0.112 |
| ***E. eneidae*** |  |  |  |  |  |
| Occupancy(elevation^2^) | 3 | 42.242 | 48.8 | 0 | 0.512 |
| Occupancy(.) | 1 | 47.532 | 49.6 | 0.86 | 0.333 |
| Occupancy(elevation) | 2 | 46.884 | 51.1 | 2.38 | 0.156 |
| ***E*. *karlschmidti*** |  |  |  |  |  |
| Occupancy(elevation^2^) | 3 | 35.576 | 42.1 | 0 | 0.497 |
| Occupancy(.) | 1 | 40.794 | 42.9 | 0.79 | 0.335 |
| Occupancy(elevation) | 2 | 40 | 44.2 | 2.16 | 0.168 |
| ***E*. *coqui*** |  |  |  |  |  |
| Occupancy(.) | 3 | 70.682 | 72.8 | 0 | 0.642 |
| Occupancy(elevation) | 1 | 70.68 | 74.9 | 2.17 | 0.217 |
| Occupancy(elevation^2^) | 2 | 69.278 | 75.8 | 3.03 | 0.141 |
