# Supplementary material for: Lowland extirpation of anuran populations on a tropical mountain
Source: PeerJ. 2017 Nov 15;5:e4059. doi: 10.7717/peerj.4059 (PMC5694215; doi:10.7717/peerj.4059)
Supplement: Table S7 — Given are the number of parameters (#Par); twice the negative log-likelihhod (AIC); the relative difference in AIC values compared to the top-ranked model (ΔAIC); the AIC model weights (AICwt); and the cumulative weights (CumWt). [file peerj-05-4059-s008.docx]

Table S7. Summary of model selection procedure for the contemporary dataset of 11 species of native frogs in the Luquillo Mountains, Puerto Rico. Given are detection probability (p), the number of parameters (K); twice the negative log-likelihhod (-2loglike); Akaike Information Criteria (AIC); the relative difference in AIC values compared to the top-ranked model (∆AIC) and the AIC model weights (AICwt)

| ***E. brittoni*** |  |  |  |  |  |
| --- | --- | --- | --- | --- | --- |
| **Model** | **K** | **-2logLike** | **AIC** | **∆AIC** | **AICwt** |
| Occupancy(elevation2) p(.) | 4 | 412.75 | 420.75 | 0 | 0.54 |
| Occupancy(elevation2) p(elevation) | 5 | 412.49 | 422.49 | 1.74 | 0.23 |
| Occupancy(elevation2) p(elevation2) | 6 | 412.24 | 424.24 | 3.48 | 0.10 |
| Occupancy(elevation) p(.) | 3 | 418.51 | 424.51 | 3.76 | 0.08 |
| Occupancy(elevation) p(elevation) | 4 | 418.25 | 426.25 | 5.49 | 0.04 |
| Occupancy(elevation) p(elevation2) | 5 | 417.99 | 427.99 | 7.24 | 0.02 |
| Occupancy(.) p(.) | 2 | 446.89 | 450.89 | 30.13 | 0.00 |
| Occupancy(.) p(elevation) | 3 | 446.62 | 452.62 | 31.87 | 0.00 |
| Occupancy(.) p(elevation2) | 4 | 446.35 | 454.35 | 33.6 | 0.00 |

***E. locustus***

| Occupancy(.) p(elevation) | 3 | 66.96 | 72.93 | 0 | 0.53 |
| --- | --- | --- | --- | --- | --- |
| Occupancy(elevation) p(elevation) | 4 | 66.58 | 74.28 | 1.35 | 0.27 |
| Occupancy(elevation^2^) p(elevation) | 5 | 66.97 | 76.33 | 3.4 | 0.10 |
| Occupancy(elevation) p(elevation^2^) | 5 | 66.53 | 76.72 | 3.79 | 0.08 |
| Occupancy(.) p(elevation^2^) | 4 | 66.62 | 79.36 | 6.43 | 0.02 |
| Occupancy(elevation^2^) p(elevation^2^) | 6 | 65.23 | 81.42 | 8.49 | 0.01 |
| Occupancy(elevation) p(.) | 3 | 103.30 | 109.3 | 36.37 | 0.00 |
| Occupancy(.) p(.) | 2 | 106.96 | 110.96 | 38.04 | 0.00 |
| Occupancy(elevation^2^) p(.) | 4 | 103.02 | 111.02 | 38.1 | 0.00 |

***E. gryllus***

| Occupancy(elevation) p(elevation^2^) | 5 | | 158.17 | 168.17 | 0 | 0.65 |  |
| --- | --- | --- | --- | --- | --- | --- | --- |
| Occupancy(elevation^2^) p(elevation^2^) | | 6 | 157.45 | 169.45 | 1.28 | 0.35 |  |
| Occupancy(.) p(elevation^2^) | 4 | | 179.22 | 187.22 | 19.05 | 0.00 |  |
| Occupancy(elevation) p(elevation) | 4 | | 209.28 | 217.28 | 49.12 | 0.00 |  |
| Occupancy(elevation^2^) p(elevation) | 5 | | 208.61 | 218.61 | 50.44 | 0.00 |  |
| Occupancy(elevation) p(.) | 3 | | 221.71 | 227.71 | 59.54 | 0.00 |  |
| Occupancy(elevation^2^) p(.) | 4 | | 221.01 | 229.01 | 60.84 | 0.00 |  |
| Occupancy(.) p(elevation) | 3 | | 226.53 | 232.53 | 64.36 | 0.00 |  |
| Occupancy(.) p(.) | 2 | | 242.72 | 246.72 | 78.55 | 0.00 |  |

***E. richmondi***

| **Model** | **K** | **-2logLike** | **AIC** | **∆AIC** | **AICwt** |
| --- | --- | --- | --- | --- | --- |
| Occupancy(elevation^2^) p(.) | 4 | 20.22 | 28.15 | 0 | 0.5724 |
| Occupancy(elevation^2^) p(elevation) | 5 | 20.19 | 30.26 | 2.11 | 0.1992 |
| Occupancy(elevation^2^) p(elevation^2^) | 6 | 20.14 | 32.16 | 4.01 | 0.0771 |
| Occupancy(elevation) p(elevation) | 4 | 27.97 | 33.45 | 5.3 | 0.0404 |
| Occupancy(elevation) p(elevation^2^) | 5 | 30.30 | 33.69 | 5.55 | 0.0358 |
| Occupancy(.) p(elevation) | 3 | 27.01 | 33.89 | 5.74 | 0.0324 |
| Occupancy(.) p(.) | 2 | 29.85 | 34.3 | 6.16 | 0.0264 |
| Occupancy(elevation) p(.) | 3 | 27.99 | 35.86 | 7.71 | 0.0121 |
| Occupancy(.) p(elevation^2^) | 4 | 28.15 | 37.97 | 9.83 | 0.0042 |

***E. wightmanae***

| Occupancy(.) p(.) | 2 | 189.14 | 193.14 | 0 | 0.271 |
| --- | --- | --- | --- | --- | --- |
| Occupancy(.) p(elevation^2^) | 4 | 185.64 | 193.64 | 0.5 | 0.211 |
| Occupancy(.) p(elevation) | 3 | 188.80 | 194.8 | 1.66 | 0.118 |
| Occupancy(elevation) p(.) | 3 | 188.88 | 194.88 | 1.75 | 0.113 |
| Occupancy(elevation) p(elevation^2^) | 5 | 185.39 | 195.39 | 2.25 | 0.088 |
| Occupancy(elevation^2^) p(.) | 4 | 187.93 | 195.93 | 2.8 | 0.067 |
| Occupancy(elevation^2^) p(elevation^2^) | 6 | 184.44 | 196.44 | 3.3 | 0.052 |
| Occupancy(elevation) p(elevation) | 4 | 188.54 | 196.54 | 3.4 | 0.049 |
| Occupancy(elevation^2^) p(elevation) | 5 | 187.59 | 197.59 | 4.45 | 0.029 |

***E. portoricensis***

| Occupancy(elevation) p(elevation^2^) | 5 | 178.26 | 188.26 | 0 | 0.50 |
| --- | --- | --- | --- | --- | --- |
| Occupancy(elevation^2^) p(elevation^2^) | 6 | 176.28 | 188.28 | 0.017 | 0.50 |
| Occupancy(elevation) p(elevation) | 4 | 192.91 | 200.91 | 12.646 | 0.00 |
| Occupancy(elevation^2^) p(elevation) | 5 | 190.92 | 200.92 | 12.663 | 0.00 |
| Occupancy(elevation) p(.) | 3 | 206.49 | 212.49 | 24.227 | 0.00 |
| Occupancy(elevation^2^) p(.) | 4 | 204.50 | 212.5 | 24.243 | 0.00 |
| Occupancy(.) p(elevation^2^) | 4 | 234.01 | 242.01 | 53.752 | 0.00 |
| Occupancy(.) p(elevation) | 3 | 248.66 | 254.66 | 66.399 | 0.00 |
| Occupancy(.) p(.) | 2 | 262.24 | 266.24 | 77.979 | 0.00 |

***E. unicolor***

| **Model** | **K** | **-2logLike** | **AIC** | **∆AIC** | **AICwt** |
| --- | --- | --- | --- | --- | --- |
| Occupancy(elevation) p(elevation) | 4 | 176.38 | 184.38 | 0 | 0.31 |
| Occupancy(elevation^2^) p(elevation) | 5 | 174.70 | 184.7 | 0.32 | 0.27 |
| Occupancy(elevation) p(elevation^2^) | 5 | 174.97 | 184.97 | 0.58 | 0.23 |
| Occupancy(elevation^2^) p(elevation^2^) | 6 | 173.48 | 185.48 | 1.09 | 0.18 |
| Occupancy(.) p(elevation^2^) | 4 | 192.68 | 200.68 | 16.3 | 0.00 |
| Occupancy(elevation) p(.) | 3 | 199.05 | 205.05 | 20.67 | 0.00 |
| Occupancy(elevation^2^) p(.) | 4 | 197.36 | 205.36 | 20.98 | 0.00 |
| Occupancy(.) p(elevation) | 3 | 212.20 | 218.2 | 33.82 | 0.00 |
| Occupancy(.) p(.) | 2 | 259.88 | 263.88 | 79.49 | 0.00 |
| ***E. hedricki*** |  |  |  |  |  |
| Occupancy(elevation^2^) p(elevation) | 5 | 383.67 | 393.67 | 0 | 0.53 |
| Occupancy(elevation^2^) p(elevation^2^) | 6 | 381.93 | 393.93 | 0.26 | 0.47 |
| Occupancy(.) p(elevation^2^) | 4 | 405.73 | 413.73 | 20.06 | 0.00 |
| Occupancy(elevation) p(elevation^2^) | 5 | 405.21 | 415.21 | 21.54 | 0.00 |
| Occupancy(elevation) p(elevation) | 4 | 408.96 | 416.96 | 23.29 | 0.00 |
| Occupancy(elevation^2^) p(.) | 4 | 409.66 | 417.66 | 24 | 0.00 |
| Occupancy(.) p(elevation) | 3 | 412.37 | 418.37 | 24.7 | 0.00 |
| Occupancy(elevation) p(.) | 3 | 435.12 | 441.12 | 47.45 | 0.00 |
| Occupancy(.) p(.) | 2 | 438.77 | 442.77 | 49.1 | 0.00 |

***E. antillensis***

| Occupancy(elevation) p(.) | 3 | 72.41 | 78.42 | 0 | 0.3482 |
| --- | --- | --- | --- | --- | --- |
| Occupancy(elevation) p(elevation) | 4 | 70.99 | 79.04 | 0.63 | 0.2547 |
| Occupancy(elevation^2^) p(.) | 4 | 72.42 | 80.41 | 1.99 | 0.1284 |
| Occupancy(elevation) p(elevation^2^) | 5 | 70.82 | 80.92 | 2.5 | 0.0997 |
| Occupancy(elevation^2^) p(elevation) | 5 | 71.00 | 81.03 | 2.62 | 0.0941 |
| Occupancy(elevation^2^) p(elevation^2^) | 6 | 71.63 | 81.83 | 3.42 | 0.0631 |
| Occupancy(.) p(elevation^2^) | 4 | 79.28 | 86.21 | 7.79 | 0.0071 |
| Occupancy(.) p(.) | 2 | 84.16 | 88.16 | 9.74 | 0.0027 |
| Occupancy(.) p(elevation) | 3 | 82.71 | 88.71 | 10.3 | 0.002 |

***E. cochranae***

| **Model** | **K** | **-2logLike** | **AIC** | **∆AIC** | **AICwt** |
| --- | --- | --- | --- | --- | --- |
| Occupancy(elevation) p(.) | 3 | 11.77 | 17.78 | 0 | 0.367 |
| Occupancy(elevation^2^) p(.) | 4 | 10.65 | 19.12 | 1.35 | 0.187 |
| Occupancy(elevation) p(elevation) | 4 | 11.71 | 19.77 | 2 | 0.135 |
| Occupancy(.) p(elevation) | 3 | 10.66 | 20.07 | 2.29 | 0.117 |
| Occupancy(elevation^2^) p(elevation) | 5 | 11.77 | 21.14 | 3.36 | 0.068 |
| Occupancy(elevation) p(elevation^2^) | 5 | 17.99 | 21.76 | 3.98 | 0.05 |
| Occupancy(.) p(.) | 2 | 10.28 | 21.99 | 4.21 | 0.045 |
| Occupancy(elevation^2^) p(elevation^2^) | 6 | 17.99 | 23.87 | 6.09 | 0.017 |
| Occupancy(.) p(elevation^2^) | 4 | 16.27 | 24.3 | 6.52 | 0.014 |

***L. albilabris***

| Occupancy(elevation) p(elevation^2^) | 5 | 361.45 | 371.45 | 0 | 0.49 |
| --- | --- | --- | --- | --- | --- |
| Occupancy(.) p(elevation^2^) | 4 | 364.23 | 372.23 | 0.78 | 0.33 |
| Occupancy(elevation^2^) p(elevation^2^) | 6 | 361.40 | 373.4 | 1.95 | 0.18 |
| Occupancy(elevation) p(elevation) | 4 | 375.66 | 383.66 | 12.21 | 0.00 |
| Occupancy(elevation^2^) p(elevation) | 5 | 374.98 | 384.98 | 13.53 | 0.00 |
| Occupancy(elevation) p(.) | 3 | 380.36 | 386.36 | 14.91 | 0.00 |
| Occupancy(elevation^2^) p(.) | 4 | 379.66 | 387.66 | 16.21 | 0.00 |
| Occupancy(.) p(elevation) | 3 | 382.31 | 388.31 | 16.86 | 0.00 |
| Occupancy(.) p(.) | 2 | 386.91 | 390.91 | 19.45 | 0.00 |
